# Diaqua­bis(5-carb­oxy-2-propyl-1*H*-imidazole-4-carboxyl­ato-κ^2^ *N* ^3^,*O* ^4^)manganese(II) *N*,*N*-dimethyl­formamide disolvate

**DOI:** 10.1107/S1600536809054634

**Published:** 2009-12-24

**Authors:** Jian-Bin Yan, Shi-Jie Li, Wen-Dong Song, Hao Wang, Dong-Liang Miao

**Affiliations:** a College of Science, Guang Dong Ocean University, Zhanjiang 524088, People’s Republic of China

## Abstract

In the title complex, [Mn(C_8_H_9_N_2_O_4_)_2_(H_2_O)_2_]·2C_3_H_7_NO, the Mn^II^ atom, lying on an inversion centre, is six-coordinated by two *N*,*O*-bidentate 5-carb­oxy-2-propyl-1*H*-imidazole-4-carb­oxyl­ate ligands and two water mol­ecules in a distorted octa­hedral environment. In the crystal structure, the complex mol­ecules and dimethyl­formamide solvent mol­ecules are linked by N—H⋯O and O—H⋯O hydrogen bonds into a two-dimensional supra­molecular network parallel to (001).

## Related literature

For the potential uses and diverse structural types of complexes containing metals and *N*-heterocyclic carboxylic acids, see: Liang *et al.* (2002 ▶); Net *et al.* (1989 ▶); Nie *et al.* (2007 ▶); Song *et al.* (2010 ▶).
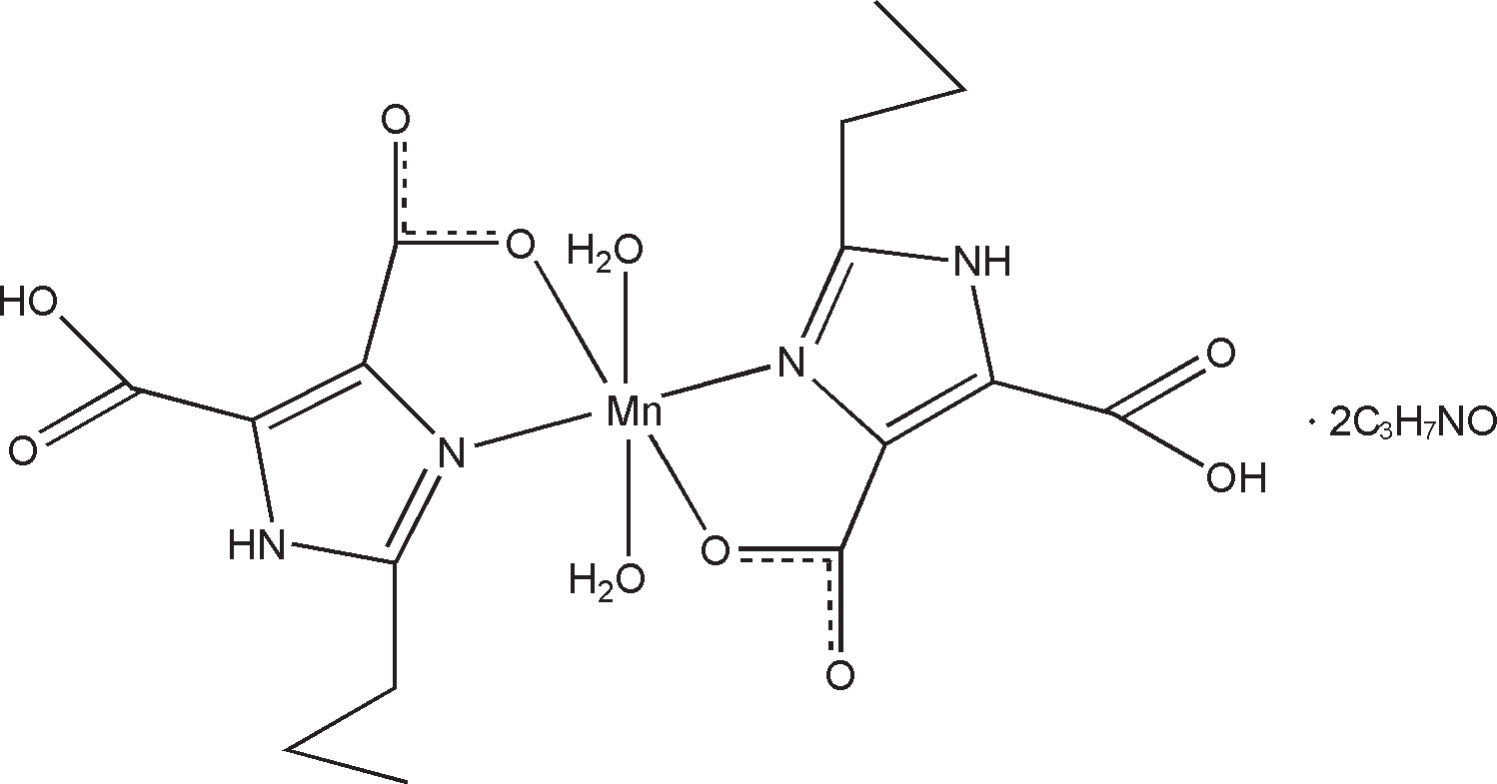


## Experimental

### 

#### Crystal data

[Mn(C_8_H_9_N_2_O_4_)_2_(H_2_O)_2_]·2C_3_H_7_NO
*M* *_r_* = 631.51
Triclinic, 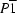

*a* = 7.3992 (8) Å
*b* = 9.4429 (11) Å
*c* = 11.1978 (13) Å
α = 76.591 (1)°
β = 87.927 (1)°
γ = 68.863 (1)°
*V* = 708.89 (14) Å^3^
*Z* = 1
Mo *K*α radiation
μ = 0.54 mm^−1^
*T* = 273 K
0.32 × 0.25 × 0.21 mm

#### Data collection

Bruker APEXII CCD diffractometer
Absorption correction: multi-scan (*SADABS*; Sheldrick, 1996 ▶)) *T*_min_ = 0.847, *T*_max_ = 0.896
3653 measured reflections
2508 independent reflections
2131 reflections with *I* > 2σ(*I*)
*R*_int_ = 0.025

#### Refinement

*R*[*F* ^2^ > 2σ(*F* ^2^)] = 0.041
*wR*(*F* ^2^) = 0.114
*S* = 1.05
2508 reflections
191 parameters
27 restraints
H-atom parameters constrained
Δρ_max_ = 0.34 e Å^−3^
Δρ_min_ = −0.32 e Å^−3^

### 

Data collection: *APEX2* (Bruker, 2007 ▶); cell refinement: *SAINT* (Bruker, 2007 ▶); data reduction: *SAINT*; program(s) used to solve structure: *SHELXS97* (Sheldrick, 2008 ▶); program(s) used to refine structure: *SHELXL97* (Sheldrick, 2008 ▶); molecular graphics: *SHELXTL* (Sheldrick, 2008 ▶); software used to prepare material for publication: *SHELXTL*.

## Supplementary Material

Crystal structure: contains datablocks I, global. DOI: 10.1107/S1600536809054634/hy2265sup1.cif

Structure factors: contains datablocks I. DOI: 10.1107/S1600536809054634/hy2265Isup2.hkl

Additional supplementary materials:  crystallographic information; 3D view; checkCIF report

## Figures and Tables

**Table 1:** Selected bond lengths (Å)

Mn1—N1	2.1960 (18)
Mn1—O1*W*	2.2036 (17)
Mn1—O1	2.2530 (17)

**Table 2:** Hydrogen-bond geometry (Å, °)

*D*—H⋯*A*	*D*—H	H⋯*A*	*D*⋯*A*	*D*—H⋯*A*
N2—H2⋯O9^i^	0.86	1.84	2.682 (3)	165
O3—H3⋯O2	0.82	1.65	2.471 (2)	176
O1*W*—H1*W*⋯O4^ii^	0.85	1.92	2.764 (2)	170
O1*W*—H2*W*⋯O4^iii^	0.84	2.11	2.927 (2)	164

Symmetry codes: (i) 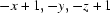; (ii) 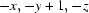; (iii) 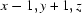.

**Fig. 1.**
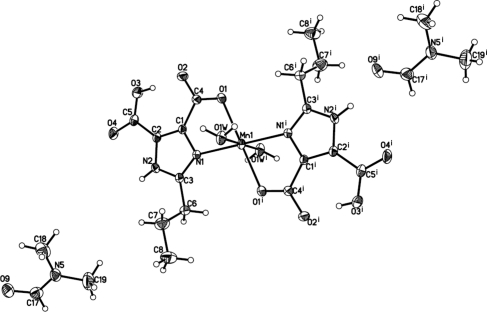
Molecular structure of the title compound. Displacement ellipsoids are shown at the 30% probability level. [Symmetry code: (i) -x, 2 - y, -z.]

**Fig. 2.**
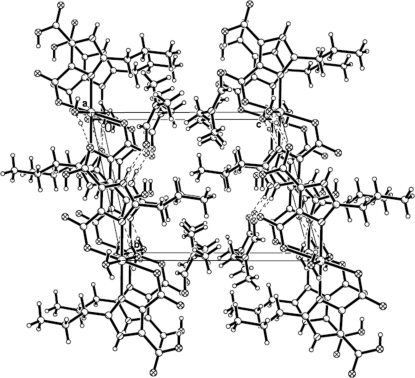
A view of the two-dimensional network constructed by O—H···O and N—H···O hydrogen bonds (dashed lines).

## supplementary crystallographic information

### Comment

Structures of complexes containing metals and N-heterocyclic carboxylic acids have attracted much attention. The N-heterocyclic carboxylic acids can function as multidentate ligands, exhibiting diverse structrual types, and their metal complexes can be potentially used as functional materials (Liang *et al.*, 2002; Net *et al.*, 1989; Nie *et al.*, 2007). Recently, we have reported a new complex, poly[diaquabis(4-carboxy-2-propyl-1*H*-imidazole-5-carboxylato- κ^3^*N*^3^,*O*^4^:*O*^5^)calcium(II)] (Song *et al.*, 2010). In this paper, we report the synthesis and structure of a Mn^II^ complex obtained under hydrothermal conditions.

As illustrated in Fig. 1, the title complex molecule contains one Mn^II^ atom, lying on an inversion centre, one mono-deprotonated 5-carboxy-2-propyl-1*H*-imidazole-4-carboxylate ligand, one coordinated water molecule and one dimethylformamide solvent molecule in the asymmetric unit. The Mn^II^ atom is six-coordinated by two N,*O*-bidentate ligands and two water molecules in a distorted octahedral environment (Table 1). In the crystal structure, a two-dimensional supramolecular network is formed by N—H···O and O—H···O hydrogen bonds (Table 2 and Fig. 2).

### Experimental

A mixture of MnCl_2_ (0.5 mmol, 0.06 g) and 2-propyl-1*H*-imidazole-4,5-dicarboxylic acid (0.5 mmol, 0.99 g) in 15 ml of dimethylformamide solution was sealed in an autoclave equipped with a Teflon liner (20 ml) and then heated at 433 K for 4 d. Crystals of the title compound were obtained by slow evaporation of the solvent at room temperature.

### Refinement

C– and N-bound H atoms were placed at calculated positions and refined as riding atoms, with C—H = 0.93 (CH), 0.97 (CH_2_) and 0.96 (CH_3_) Å and N—H = 0.86 Å and with *U*_iso_(H) = 1.2(1.5 for methyl)*U*_eq_(C, N). H atoms of water and carboxyl group were located in a difference Fourier map and refined as riding atoms, with *U*_iso_(H) = 1.5*U*_eq_(O).

### Crystal data

**Table:**

[Mn(C_8_H_9_N_2_O_4_)_2_H_2_O)_2_]·2C_3_H_7_NO	*Z* = 1
*M**_r_* = 631.51	*F*(000) = 331
Triclinic, *P*1	*D*_x_ = 1.479 Mg m^−^^3^
Hall symbol: -P 1	Mo *K*α radiation, λ = 0.71073 Å
*a* = 7.3992 (8) Å	Cell parameters from 3600 reflections
*b* = 9.4429 (11) Å	θ = 1.4–28°
*c* = 11.1978 (13) Å	µ = 0.54 mm^−^^1^
α = 76.591 (1)°	*T* = 273 K
β = 87.927 (1)°	Block, colourless
γ = 68.863 (1)°	0.32 × 0.25 × 0.21 mm
*V* = 708.89 (14) Å^3^	

### Data collection

**Table:**

Bruker APEXII CCD diffractometer	2131 reflections with *I* > 2σ(*I*)
Radiation source: fine-focus sealed tube	*R*_int_ = 0.025
φ and ω scan	θ_max_ = 25.2°, θ_min_ = 1.9°
Absorption correction: multi-scan (*SADABS*; Sheldrick, 1996))	*h* = −8→8
*T*_min_ = 0.847, *T*_max_ = 0.896	*k* = −9→11
3653 measured reflections	*l* = −13→12
2508 independent reflections	

### Refinement

**Table:**

Refinement on *F*^2^	Primary atom site location: structure-invariant direct methods
Least-squares matrix: full	Secondary atom site location: difference Fourier map
*R*[*F*^2^ > 2σ(*F*^2^)] = 0.041	Hydrogen site location: inferred from neighbouring sites
*wR*(*F*^2^) = 0.114	H-atom parameters constrained
*S* = 1.05	*w* = 1/[σ^2^(*F*_o_^2^) + (0.0549*P*)^2^ + 0.120*P*] where *P* = (*F*_o_^2^ + 2*F*_c_^2^)/3
2508 reflections	(Δ/σ)_max_ < 0.001
191 parameters	Δρ_max_ = 0.34 e Å^−^^3^
27 restraints	Δρ_min_ = −0.32 e Å^−^^3^

### Fractional atomic coordinates and isotropic or equivalent isotropic displacement parameters (Å^2^)

**Table:**

	*x*	*y*	*z*	*U*_iso_*/*U*_eq_	
Mn1	0.0000	1.0000	0.0000	0.03110 (18)	
O1	−0.0548 (3)	0.92199 (19)	−0.16620 (15)	0.0379 (4)	
O1W	−0.2795 (2)	0.9893 (2)	0.06711 (17)	0.0434 (4)	
H1W	−0.2933	0.9113	0.0484	0.065*	
H2W	−0.3672	1.0743	0.0329	0.065*	
O2	0.0012 (3)	0.7160 (2)	−0.24509 (15)	0.0424 (4)	
O3	0.1886 (3)	0.4324 (2)	−0.18355 (17)	0.0469 (5)	
H3	0.1294	0.5271	−0.2021	0.070*	
O4	0.3630 (3)	0.25664 (19)	−0.02155 (18)	0.0456 (5)	
N1	0.1434 (3)	0.7447 (2)	0.04969 (16)	0.0279 (4)	
N2	0.3073 (3)	0.4924 (2)	0.10599 (17)	0.0312 (4)	
H2	0.3782	0.4030	0.1504	0.037*	
C1	0.1335 (3)	0.6770 (2)	−0.0454 (2)	0.0261 (5)	
C2	0.2340 (3)	0.5196 (3)	−0.0111 (2)	0.0285 (5)	
C3	0.2498 (3)	0.6292 (3)	0.1400 (2)	0.0303 (5)	
C4	0.0194 (3)	0.7798 (3)	−0.1592 (2)	0.0306 (5)	
C5	0.2661 (3)	0.3927 (3)	−0.0755 (2)	0.0337 (5)	
C6	0.2933 (4)	0.6449 (3)	0.2642 (2)	0.0410 (6)	
H6A	0.2584	0.7548	0.2625	0.049*	
H6B	0.4317	0.5939	0.2840	0.049*	
C7	0.1855 (5)	0.5745 (4)	0.3637 (3)	0.0616 (8)	
H7A	0.0477	0.6206	0.3409	0.074*	
H7B	0.2268	0.4633	0.3688	0.074*	
C8	0.2178 (5)	0.5986 (4)	0.4887 (3)	0.0648 (9)	
H8A	0.3457	0.5307	0.5217	0.097*	
H8B	0.1234	0.5753	0.5427	0.097*	
H8C	0.2049	0.7052	0.4811	0.097*	
O9	0.4607 (3)	−0.2429 (2)	0.7294 (2)	0.0626 (6)	
N5	0.3789 (3)	0.0120 (3)	0.6354 (2)	0.0470 (6)	
C17	0.4889 (4)	−0.1190 (4)	0.7092 (3)	0.0501 (7)	
H17	0.5962	−0.1181	0.7492	0.060*	
C18	0.2038 (5)	0.0185 (4)	0.5761 (3)	0.0702 (10)	
H18A	0.0937	0.0641	0.6212	0.105*	
H18B	0.2138	−0.0852	0.5745	0.105*	
H18C	0.1880	0.0810	0.4936	0.105*	
C19	0.4104 (7)	0.1561 (4)	0.6248 (4)	0.0879 (12)	
H19A	0.5309	0.1353	0.6673	0.132*	
H19B	0.3061	0.2259	0.6604	0.132*	
H19C	0.4151	0.2033	0.5396	0.132*	

### Atomic displacement parameters (Å^2^)

**Table:**

	*U*^11^	*U*^22^	*U*^33^	*U*^12^	*U*^13^	*U*^23^
Mn1	0.0369 (3)	0.0180 (3)	0.0364 (3)	−0.0063 (2)	−0.0014 (2)	−0.0080 (2)
O1	0.0483 (10)	0.0222 (9)	0.0367 (9)	−0.0057 (8)	−0.0087 (7)	−0.0043 (7)
O1W	0.0411 (10)	0.0287 (9)	0.0616 (12)	−0.0120 (8)	0.0038 (8)	−0.0142 (8)
O2	0.0568 (11)	0.0346 (10)	0.0329 (9)	−0.0101 (9)	−0.0099 (8)	−0.0112 (8)
O3	0.0609 (12)	0.0307 (10)	0.0473 (11)	−0.0079 (9)	−0.0035 (9)	−0.0189 (8)
O4	0.0465 (10)	0.0218 (9)	0.0645 (12)	−0.0044 (8)	−0.0026 (9)	−0.0145 (8)
N1	0.0344 (10)	0.0210 (10)	0.0277 (10)	−0.0092 (8)	−0.0017 (8)	−0.0055 (8)
N2	0.0332 (10)	0.0188 (9)	0.0351 (11)	−0.0048 (8)	−0.0052 (8)	−0.0002 (8)
C1	0.0279 (11)	0.0210 (11)	0.0294 (11)	−0.0085 (9)	0.0007 (9)	−0.0064 (9)
C2	0.0288 (11)	0.0228 (12)	0.0341 (12)	−0.0087 (9)	0.0016 (9)	−0.0083 (9)
C3	0.0340 (12)	0.0234 (12)	0.0328 (12)	−0.0106 (10)	−0.0036 (9)	−0.0040 (9)
C4	0.0330 (12)	0.0272 (12)	0.0295 (12)	−0.0087 (10)	−0.0024 (9)	−0.0055 (10)
C5	0.0326 (12)	0.0267 (13)	0.0440 (14)	−0.0105 (10)	0.0066 (10)	−0.0138 (11)
C6	0.0512 (15)	0.0365 (14)	0.0354 (13)	−0.0164 (12)	−0.0098 (11)	−0.0062 (11)
C7	0.072 (2)	0.076 (2)	0.0455 (16)	−0.0325 (18)	0.0089 (15)	−0.0238 (15)
C8	0.069 (2)	0.077 (2)	0.0424 (16)	−0.0168 (19)	0.0026 (15)	−0.0191 (16)
O9	0.0712 (14)	0.0298 (11)	0.0699 (14)	−0.0062 (10)	−0.0216 (11)	0.0047 (10)
N5	0.0535 (13)	0.0309 (12)	0.0502 (13)	−0.0098 (11)	0.0019 (11)	−0.0065 (10)
C17	0.0472 (15)	0.0471 (18)	0.0500 (16)	−0.0084 (14)	−0.0051 (13)	−0.0129 (13)
C18	0.0575 (19)	0.058 (2)	0.073 (2)	−0.0073 (16)	−0.0120 (16)	0.0061 (17)
C19	0.134 (4)	0.050 (2)	0.089 (3)	−0.044 (2)	0.019 (3)	−0.0182 (19)

### Geometric parameters (Å, °)

**Table:**

Mn1—N1	2.1960 (18)	C6—H6A	0.9700
Mn1—O1W	2.2036 (17)	C6—H6B	0.9700
Mn1—O1	2.2530 (17)	C7—C8	1.509 (4)
O1—C4	1.238 (3)	C7—H7A	0.9700
O1W—H1W	0.8509	C7—H7B	0.9700
O1W—H2W	0.8436	C8—H8A	0.9600
O2—C4	1.282 (3)	C8—H8B	0.9600
O3—C5	1.272 (3)	C8—H8C	0.9600
O3—H3	0.8200	O9—C17	1.229 (4)
O4—C5	1.240 (3)	N5—C17	1.313 (4)
N1—C3	1.326 (3)	N5—C19	1.440 (4)
N1—C1	1.379 (3)	N5—C18	1.452 (4)
N2—C3	1.347 (3)	C17—H17	0.9300
N2—C2	1.369 (3)	C18—H18A	0.9600
N2—H2	0.8600	C18—H18B	0.9600
C1—C2	1.367 (3)	C18—H18C	0.9600
C1—C4	1.480 (3)	C19—H19A	0.9600
C2—C5	1.481 (3)	C19—H19B	0.9600
C3—C6	1.491 (3)	C19—H19C	0.9600
C6—C7	1.516 (4)		
			
N1^i^—Mn1—N1	180.0	O4—C5—C2	118.9 (2)
N1^i^—Mn1—O1W	87.40 (7)	O3—C5—C2	116.6 (2)
N1—Mn1—O1W	92.60 (6)	C3—C6—C7	112.7 (2)
N1^i^—Mn1—O1W^i^	92.60 (6)	C3—C6—H6A	109.1
N1—Mn1—O1W^i^	87.40 (7)	C7—C6—H6A	109.1
O1W—Mn1—O1W^i^	180.0	C3—C6—H6B	109.1
N1^i^—Mn1—O1^i^	75.41 (6)	C7—C6—H6B	109.1
N1—Mn1—O1^i^	104.59 (6)	H6A—C6—H6B	107.8
O1W—Mn1—O1^i^	91.40 (6)	C8—C7—C6	113.4 (3)
O1W^i^—Mn1—O1^i^	88.60 (6)	C8—C7—H7A	108.9
N1^i^—Mn1—O1	104.59 (6)	C6—C7—H7A	108.9
N1—Mn1—O1	75.41 (6)	C8—C7—H7B	108.9
O1W—Mn1—O1	88.60 (6)	C6—C7—H7B	108.9
O1W^i^—Mn1—O1	91.40 (6)	H7A—C7—H7B	107.7
O1^i^—Mn1—O1	180.0	C7—C8—H8A	109.5
C4—O1—Mn1	115.45 (14)	C7—C8—H8B	109.5
Mn1—O1W—H1W	107.7	H8A—C8—H8B	109.5
Mn1—O1W—H2W	107.5	C7—C8—H8C	109.5
H1W—O1W—H2W	112.0	H8A—C8—H8C	109.5
C5—O3—H3	109.5	H8B—C8—H8C	109.5
C3—N1—C1	105.96 (18)	C17—N5—C19	121.9 (3)
C3—N1—Mn1	141.35 (15)	C17—N5—C18	119.5 (3)
C1—N1—Mn1	112.53 (13)	C19—N5—C18	118.0 (3)
C3—N2—C2	108.51 (18)	O9—C17—N5	125.0 (3)
C3—N2—H2	125.7	O9—C17—H17	117.5
C2—N2—H2	125.7	N5—C17—H17	117.5
C2—C1—N1	109.72 (19)	N5—C18—H18A	109.5
C2—C1—C4	132.5 (2)	N5—C18—H18B	109.5
N1—C1—C4	117.81 (19)	H18A—C18—H18B	109.5
C1—C2—N2	105.37 (19)	N5—C18—H18C	109.5
C1—C2—C5	132.1 (2)	H18A—C18—H18C	109.5
N2—C2—C5	122.5 (2)	H18B—C18—H18C	109.5
N1—C3—N2	110.44 (19)	N5—C19—H19A	109.5
N1—C3—C6	125.5 (2)	N5—C19—H19B	109.5
N2—C3—C6	124.0 (2)	H19A—C19—H19B	109.5
O1—C4—O2	123.4 (2)	N5—C19—H19C	109.5
O1—C4—C1	118.6 (2)	H19A—C19—H19C	109.5
O2—C4—C1	117.9 (2)	H19B—C19—H19C	109.5
O4—C5—O3	124.4 (2)		

Symmetry codes: (i) −*x*, −*y*+2, −*z*.

### Hydrogen-bond geometry (Å, °)

**Table:**

*D*—H···*A*	*D*—H	H···*A*	*D*···*A*	*D*—H···*A*
N2—H2···O9^ii^	0.86	1.84	2.682 (3)	165
O3—H3···O2	0.82	1.65	2.471 (2)	176
O1W—H1W···O4^iii^	0.85	1.92	2.764 (2)	170
O1W—H2W···O4^iv^	0.84	2.11	2.927 (2)	164

Symmetry codes: (ii) −*x*+1, −*y*, −*z*+1; (iii) −*x*, −*y*+1, −*z*; (iv) *x*−1, *y*+1, *z*.

## References

[bb1] Bruker (2007). *APEX2* and *SAINT* Bruker AXS Inc., Madison, Wisconsin, USA.

[bb2] Liang, Y. C., Cao, R. & Hong, M. C. (2002). *Inorg. Chem. Commun.* **5**, 366–368.

[bb3] Net, G., Bayon, J. C., Butler, W. M. & Rasmussen, P. (1989). *J. Chem. Soc. Chem. Commun.* pp. 1022–1023.

[bb4] Nie, X.-L., Wen, H.-L., Wu, Z.-S., Liu, D.-B. & Liu, C.-B. (2007). *Acta Cryst.* E**63**, m753–m755.

[bb5] Sheldrick, G. M. (1996). *SADABS* University of Göttingen, Germany.

[bb6] Sheldrick, G. M. (2008). *Acta Cryst.* A**64**, 112–122. 10.1107/S0108767307043930 18156677

[bb7] Song, W.-D., Yan, J.-B., Li, S.-J., Miao, D.-L. & Li, X.-F. (2010). *Acta Cryst.* E**66**, m53.

